# The complete mitochondrial genome sequence of *Scolopendra mutilans* L. Koch, 1878 (Scolopendromorpha, Scolopendridae), with a comparative analysis of other centipede genomes

**DOI:** 10.3897/zookeys.925.47820

**Published:** 2020-04-08

**Authors:** Chaoyi Hu, Shuaibin Wang, Bisheng Huang, Hegang Liu, Lei Xu, Zhigang Hu, Yifei Liu

**Affiliations:** 1 College of Pharmacy,; 2 Hubei University of Chinese Medicine, No. 1 Huangjiahu West Road, Hongshan District, Wuhan, China; 3 Hubei Jingui Chinese Medicine Pieces Ltd., Wuhan, Hubei, China

**Keywords:** Chilopoda, Chinese medicinal materials, mitogenome, *Scolopendra mutilans*

## Abstract

*Scolopendra mutilans* L. Koch, 1878 is an important Chinese animal with thousands of years of medicinal history. However, the genomic information of this species is limited, which hinders its further application. Here, the complete mitochondrial genome (mitogenome) of *S. mutilans* was sequenced and assembled by next-generation sequencing. The genome is 15,011 bp in length, consisting of 13 protein-coding genes (PCGs), 14 tRNA genes, and two rRNA genes. Most PCGs start with the ATN initiation codon, and all PCGs have the conventional stop codons TAA and TAG. The *S. mutilans* mitogenome revealed nine simple sequence repeats (SSRs), and an obviously lower GC content compared with other seven centipede mitogenomes previously sequenced. After analysis of homologous regions between the eight centipede mitogenomes, the *S. mutilans* mitogenome further showed clear genomic rearrangements. The phylogenetic analysis of eight centipedes using 13 conserved PCG genes was finally performed. The phylogenetic reconstructions showed Scutigeromorpha as a separate group, and Scolopendromorpha in a sister-group relationship with Lithobiomorpha and Geophilomorpha. Collectively, the *S. mutilans* mitogenome provided new genomic resources, which will improve its medicinal research and applications in the future.

## Introduction

Animal medicine is an important part of the Chinese traditional medicine system. As a typical representative of medicinal animals, the centipede *Scolopendra mutilans* has been used for hundreds of years in China for treating many disorders, such as stroke-induced hemiplegia, epilepsy, apoplexy, whooping cough, tetanus, burns, tuberculosis, and myocutaneous disease (Ding et al. 2016). Moreover, centipedes have been described for the treatment of cardiovascular diseases in Korea, China, and other east Asian countries (Chen et al. 2014). *Scolopendra mutilans* is a venom-containing animal, which is rich in antimicrobial peptides, ion channel modulators, enzymes, and other macromolecular active substances (Yoo et al. 2014). Due to its active ingredients, it is of great interest in modern medical research. However, with the increase of medicinal applications, the wild populations of *S. mutilans* were over-exploited and declined greatly (Kang et al. 2017). Conservation and further artificial culture are needed, which in turn depends on the correct classification and molecular identification of the natural centipede taxa.

Centipedes (Chilopoda) are one of the oldest extant terrestrial arthropods. Approximately 3300 centipede species have been described (Chipman et al. 2014) and the majority of these taxa are distributed in tropical and subtropical regions. Six orders of centipedes are currently recognized, namely, Scolopendromorpha, Geophilomorpha, Lithobiomorpha, Scutigeromorpha, Craterostigmomorpha, and Devonobiomorpha (Bortolin et al. 2018). Devonobiomorpha is an extinct order represented by a single species (Shear and Bonamo 1988) and the Craterostigmomorpha only occur in Tasmania and New Zealand (Undheim et al. 2016). The remaining orders are distributed widely (Edgecombe et al. 2002), but their evolutionary relationships remain unclear on the basis of morphological traits. The Scutigeromorpha, with body respiratory openings on the back, was generally classified as class Notostigmophora, while the remaining orders with lateral spiracles were divided into another class, Pleurostigmophora (Giribet et al. 1999). However, both Scutigeromorpha and Lithobiomorpha have an anamorphic development in which the segment number increases during postembryonic life (Anamorpha). While Scolopendromorpha and Geophilomorpha have an epimorphic development in which the definitive number of body segments appears upon hatching (Epimorpha). The Craterostigmomorpha order is not strictly anamorphic, making its position unclear (Giribet et al. 1999; Edgecombe and Giribet 2007).

Previously, phylogenetic analysis on the basis of different molecular data provided support to these morphological classifications to some degree (Regier et al. 2008; Fernández et al. 2016). With a phylogenetic reconstruction based on a large number of protein-coding nuclear genes, the Scutigeromorpha was placed as a single evolutionary branch in Chilopoda, while the other three orders were clustered together, in which the Lithobiomorpha was a sister group of the Scolopendromorpha and the Geophilomorpha showed a distant relationship to them (Regier et al. 2008). A phylogenomic reconstruction based on transcriptomic data also suggested a similar pattern, that the Scutigeromorpha order was a sister group with the other three orders. Moreover, the Scolopendromorpha order is closer to the Geophilomorpha order than the Lithobiomorpha (Fernández et al. 2016).

The mitochondrial genome (mitogenome), including those markers derived from it as well as the whole mitogenome, is the most commonly used molecule in animal studies with relation to taxonomy, population genetics, and evolutionary biology (Wolstenholme 1992; Li et al. 2018a). Generally, an animal mitogenome is a double-stranded circular molecule, ranging from 14 to 20 kb in length and containing a typical set of 37 genes, including 13 protein-coding genes (PCGs), 22 transfer RNA (tRNA) genes, and two ribosomal RNA (rRNA) genes (Taanman 1999). Functional information on replication derived from the related genomic structures has been well investigated, but the transcription features of animal mitogenomes are still limited (Chen and Du 2017). Here, we sequenced and assembled the mitogenome of *S. mutilans* and compared its genome to seven other representative centipede mitogenomes derived from Scolopendromorpha, Geophilomorpha, Lithobiomorpha, and Scutigeromorpha. We obtained the phylogenetic relationship of these centipede taxa based on the 13 PCGs and our results provide new genetic information for both conservation and sustainable use of centipedes as a medicinal resource.

## Materials and methods

### Sample collection and DNA extraction

*Scolopendra mutilans* samples were collected in August 2018 from the wild in Yichang, Hubei Province, China. The specimens used in this study were preserved in 100% ethanol and stored at -20 °C. Genomic DNA was extracted from locomotory legs by Column mtDNAout kit (Tiangen Biotech Co., China) according to the instructions and stored at -20 °C until used for sequencing. The DNA quality was measured by gel electrophoresis and the concentration was estimated using the Nanodrop ND-1000.

### Sequencing, assembly, and annotation of mitochondrial genomes

Whole genome sequencing was performed on an Illumina HiSeq 2500 platform (Illumina, San Diego, CA, USA). Quality control and de novo assembly of the *S. mutilans* mitogenome were conducted based on previously described methods (Li et al. 2018b). Briefly, raw reads were first filtered to generate clean data. De novo assembly of mitogenomes were performed using the SPAdes v3.9.0 software package (He et al. 2018), and the gaps were filled using MITObim v1.9 (Deng et al. 2018).

The mitogenomes were annotated by combining results from both MFannot and MITOS (Bernt et al. 2013), using the genetic code 4 in both programs. The PCGs, rRNA and tRNA were initially annotated at this step. The annotated PCGs were then refined using the NCBI Open Reading Frame Finder, and further annotated with BLASTp searches against the NCBI non-redundant protein sequence database (He et al. 2018; Wang et al. 2018b). The tRNA genes were also predicted using tRNAscan-SE v1.3.1 (Zhang et al. 2018). Subsequently, graphical maps of the complete mitogenomes were drawn using OGDraw v1.2 (Wang et al. 2018a).

### Repetitive element analysis

In order to identify interspersed repeats or intra-genomic duplications of large fragments throughout the mitogenomes, we performed BLASTn searches of the mitogenome against itself using an E-value of 1e-10. Tandem repeats within the mitogenome were detected by MicroSAtellite (MISA) (Shaoli et al. 2018; Thiel et al. 2003), with the following thresholds: ten, six, five, five, five, and five repeat units for mono-nucleotide, di-nucleotide, tri-nucleotide, tetranucleotide, penta-nucleotide, and hexa-nucleotide SSRs. Forward (direct), reverse, complemented, and palindromic (reverse complemented) repeats were identified using the REPuter software (Kurtz et al. 2001) with default settings.

The base composition of the mitogenome was determined using the DNAStar Lasergene package v7.1 (Burland 2000). The following formulae were used to assess mitogenome strand asymmetry: AT skew = [A – T] / [A + T]; GC skew = [G – C] / [G + C]. Lastly, genomic synteny of the eight mitogenomes was analyzed with Mauve v2.4.0 (Darling et al. 2004).

### Phylogenetic analysis

A maximum likelihood (ML) tree was constructed using the RAxML (Stamatakis et al. 2017) based on nucleotide sequence data of 13 PCGs derived from eight centipede species among the class Chilopoda (Table 1) and a *Sphaerotheriidae* sp. (NC_018361) (Dong et al. 2012) from the class Diplopoda was used as the outgroup. The nucleotide sequences of the 13 PCGs were firstly aligned with Clustal X (Larkin et al. 2007) as implemented in MEGA7 (Kumar et al. 2008) using the default settings. The best nucleotide substitution model was determined with Jmodeltest (Posada 2008) and the GTR+G+I model was predetermined for analyses. One thousand bootstrap replicates were performed and the phylogenetic tree was illustrated using the software FigTree v1.4.2 (Lemey et al. 2010).

Analysis of selective pressures was performed for 13 PCGs of eight centipedes using the codeml program in PAML (University College London, London, UK) (Yang 2007) by calculating the nonsynonymous (*K_A_*) and synonymous (*K_S_*) substitution ratio. The method reported by Yang and Nielsen (2000) was adopted to estimate the ω value (ω = *K_A_ / K_S_*) of every gene sequence.

## Results

### Gene content and composition

**Figure 1.**
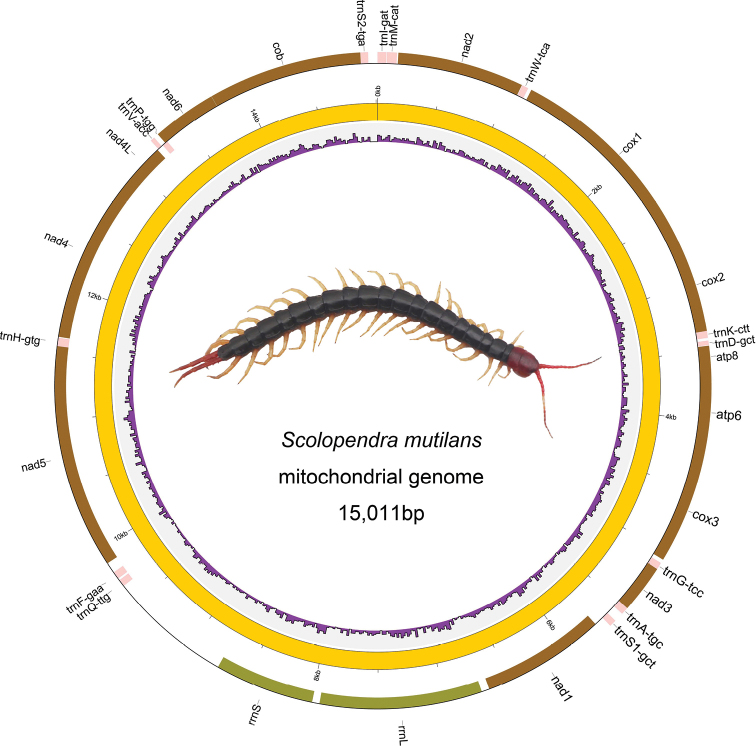
Mitochondrial genome map of the *Scolopendra mutilans*. Genes drawn inside the circle are transcribed clockwise, and those outside are counterclockwise. PCGs are shown as brown arrows, rRNA genes as green arrows, tRNA genes as pink arrows. The innermost circle shows the GC content. GC content is plotted as the deviation from the average value of the entire sequence.

The full circular mitogenome of *S. mutilans* (GenBank: MN317390) was 15,011 bp in length, which was similar to those of seven other centipede mitogenomes sequenced in the class Chilopoda (Table 1) (Robertson et al. 2015; Sun et al. 2018). The *S. mutilans* mitogenome contains 29 genes, including 13 PCGs, 14 tRNA genes, and two rRNA genes (Figure 1). Most PCGs, including the cox1, cox2, cox3, nad2, nad3, nad6, atp6, atp8, and cob genes, and the majority of tRNA genes (trnI, trnM, trnW, trnK, trnD, trnG, trnA, trnS1, trnV, and trnS2) are transcribed from the plus strand, while the remaining four PCGs, two ribosomal genes, and four tRNAs are transcribed from the minus strand (Table 2). The overlapping regions between genes were in relation to three neighboring gene pairs, containing a length of 27 bp in total, with each size ranging from 2 to 18 bp. We also found a total of 2111 bp of intergenic regions on the *S. mutilans* mitogenome, accounting for 14% of the genome size.

The mitogenome size of eight centipedes ranged from 14,538 bp for *S. dehaani* Brandt, 1840 to 16,833 bp for *Cermatobius longicornis* Takakuwa,1939, with that of *S. mutilans* in the middle of the range. To identify the specific variation contributing most to the diversity of the mitogenome size in centipedes, the length variation of all PCGs, tRNA, and rRNA genes, and intergenic regions in each mitogenome was investigated. Comparatively, the length of most genes across centipede species was relatively stable except the PCGs in *L. forficatus* Linnaeus,1758 (AF309492.1), while the length of intergenic regions was the primary contributor to mitogenome size variation.

**Table 1.** Basic information of the mitogenomes for Chilopoda used in this study.

Species	Order	NCBI ID	Length (bp)
*Scolopendra mutilans* L. Koch, 1878	Scolopendromorpha	MN317390	15011
*Scolopendra dehaani* Brandt, 1840	Scolopendromorpha	KY947341.1	14538
*Scolopocryptops* sp.	Scolopendromorpha	KC200076.1	15119
*Strigamia maritima* (Leach, 1817)	Geophilomorpha	KP173664.1	14983
*Cermatobius longicornis* Takakuwa,1939	Lithobiomorpha	NC_021403.1	16833
*Bothropolys* sp.	Lithobiomorpha	AY691655.1	15139
*Lithobius forficatus* (Linnaeus,1758)	Lithobiomorpha	AF309492.1	15695
*Scutigera coleoptrata* (Linnaeus, 1758)	Scutigeromorpha	AJ507061.2	14922

**Table 2.** Organization of the *Scolopendra mutilans* mitogenome.

Gene	Start	End	Strand	Length	Start/End codon
trnI (gat)	1	65	+	65	–
trnM (cat)	69	141	+	73	–
nad2	124	954	+	831	ATT/TAA
trnW (tca)	1071	1119	+	49	–
cox1	1148	2656	+	1509	ATG/TAG
cox2	2676	3341	+	666	ATG/TAA
trnK (ctt)	3355	3405	+	51	–
trnD (gtc)	3425	3458	+	34	–
atp8	3465	3614	+	150	ATA/TAA
atp6	3620	4267	+	648	ATG/TAA
cox3	4282	5049	+	768	AYG/TAA
trnG (tcc)	5084	5137	+	54	–
nad3	5141	5488	+	348	ATT/TAG
trnA (tgc)	5487	5542	+	56	–
trnS1 (gct)	5612	5662	+	51	–
nad1	5784	6635	-	851	ATT/TAA
rrnL	6719	7937	-	1219	–
rrnS	7993	8740	-	748	–
trnQ (ttg)	9667	9720	-	54	–
trnF (gaa)	9735	9791	-	57	–
nad5	9906	11,474	-	1569	ATT/TAA
trnH (gtg)	11,556	11,619	-	64	–
nad4	11,641	12,789	-	1149	ATG/TAA
nad4l	12,939	13,181	-	243	ATA/TAA
trnV (aac)	13,230	13,262	+	33	–
trnP (tgg)	13,272	13,315	-	44	–
nad6	13,373	13,759	+	387	ATT/TAA
cob	13,773	14,873	+	1101	ATG/TAA
trnS2 (tga)	14,889	14,944	+	56	–

### Genomic repeats

The repeated DNA in animal mitogenomes can be divided into tandem repeats and interspersed repeats (Wu et al. 2017). In the *S. mutilans* mitogenome, 46 tandem repeats have been identified, of which the longest is 39 bp and the shortest is 9 bp. However, no interspersed repeat was found. Generally, SSRs are a group of tandem repeated sequences containing 1–6 nucleotide repeat units and are widely distributed in animal mitogenomes, and they are commonly used as molecular markers for species identification (Wang et al. 2018a). A total of nine SSRs were detected in the *S. mutilans* mitogenome, including three mono-nucleotides, five di-nucleotides, and one tri-nucleotide, as well as two compound SSRs (Table 3). Among these, only one mono-nucleotide SSR is distributed in the small subunit of one ribosomal RNA gene, while the other SSRs are all presented in the intergenic regions. These mitogenomic SSRs will provide additional marker information for future genetic analyses of *S. mutilans* samples and its related species.

**Table 3.** Simple sequence repeats in *Scolopendra mutilans*.

Number	SSR type	SSR	Size (bp)	Start	End	Position
1	mono-nucleotide	(A)_11_	11	12,790	12,800	intergenic
2	mono-nucleotide	(A)_12_	12	8540	8551	rrnS
3	mono-nucleotide	(A)_20_	20	12,837	12,856	intergenic
4	di-nucleotide	(AT)_8_	16	8776	8791	intergenic
5	di-nucleotide	(AT)_8_	17	9820	9835	intergenic
6	di-nucleotide	(AT)_9_	19	3406	3423	intergenic
7	di-nucleotide	(TA)_11_	22	1119	1140	intergenic
8	di-nucleotide	(AT)_19_	39	14,968	15,005	intergenic
9	tri-nucleotide	(TAA)_5_	17	14,954	14,968	intergenic

### Protein-coding genes

**Figure 2.**
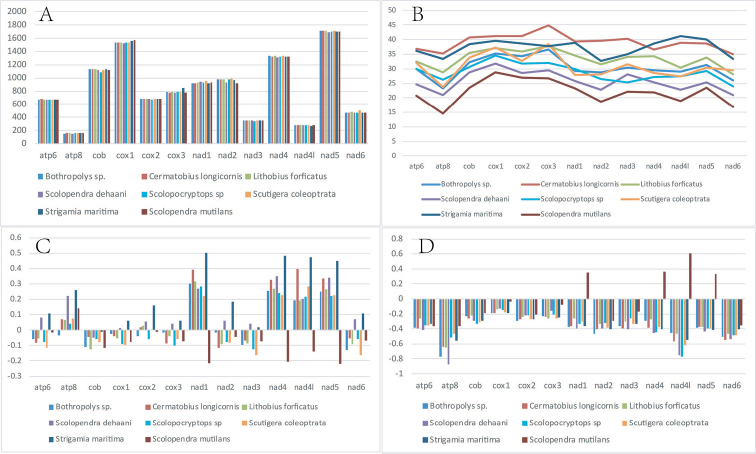
Variation in length and base composition of each of the 13 core protein coding genes (PCGs) among eight centipedes’ mitochondrial genomes **A** PCG length variation **B** GC content across PCGs**C** AT skew **D** GC skew.

For all 13 PCGs identified in the *S. mutilans* mitogenome, five genes (nad2, nad3, nad1, nad5 and nad6) initiated with the start codon ATT, two genes (atp8 and nad4l) started with the ATG codon, and the remaining six genes used ATA as the start codon. The most common termination codon TAA was detected in eleven PCGs (nad2, cox2, atp8, atp6, cox3, nad1, nad5, nad4, nad4l, nad6, cob). The cox1 and nad3 genes had termination codons with TAG (Table 2). We further compared the PCGs between different centipede mitogenomes (Table 1). Across the eight centipede mitogenomes investigated, we found that the length of some PCGs was variable; for instance, the NADH dehydrogenase genes in *S. mutilans* is a little shorter than those in other centipedes, especially for both the nad2 and nad4 genes (Figure 2A). Notably, it was found that the mean length of PCG genes in the *L. forficatus* (AF309492.1) mitogenome was slightly shorter; this may be caused by post-transcriptional editing that occurs in its mitochondrial tRNAs, which may play an important role in the synthesis of subunits of ATPase in PCGs according to previous reports (Lavrov et al. 2000). Moreover, the GC content of the 13 PCGs across these mitogenomes was also different. We found two subunits of both ATPase genes (atp6 and atp8) showed the lowest GC content compared with the other PCGs in the majority of all mitogenomes. The genetic relationship is usually positively correlated with the GC content of the mitogenome of a species (Bohlin 2011). Comparatively, we found that *S. mutilans* had the lowest GC content in all investigated species at the whole genome level, and *S. dehaani*, another species of the same genus, showed the second lowest GC content of all mitogenomes we investigated (Figure 2B). Interestingly, the four NADH dehydrogenase subunits (nad1, nad4, nad4l, nad5) possessed the opposite AT skew (Figure 2C) and GC skew in the *S. mutilans* mitogenome compared with other species (Figure 2D).

### Genomic arrangement analysis

**Figure 3.**
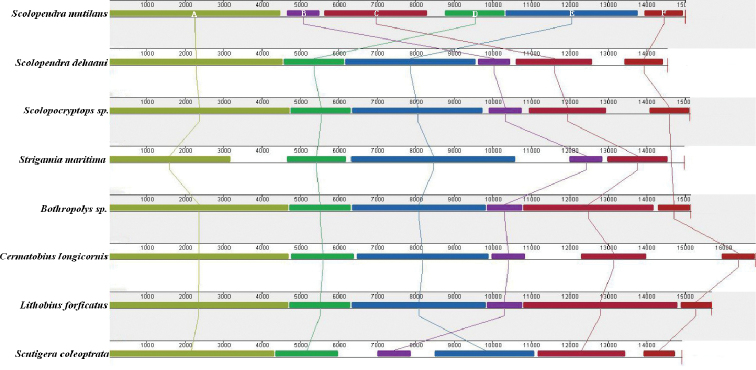
Mitogenome synteny among eight centipede species. Synteny analyses were generated in Mauve 2.4.0. A total of six large homologous regions were identified among the eight mitogenomes, while the sizes and relative positions of the homologous fragments varied across the mitogenomes.

By using the Mauve analysis, we identified six large genomic homologous regions (marked A–F in Figure 3). These homologous regions were commonly presented in all eight centipede mitogenomes, and their sequence lengths were variable across regions and genomes, particularly for the A and E regions, which had a relatively large fragmental size and greatly contributed to the genome size variation between centipede mitogenomes (Figure 3). Interestingly, we found the arrangement of these homologous regions was not conserved, particularly between the *S. mutilans* mitogenome and that of the other species (Figure 3). For example, *S. mutilans* contained a B-C-D-E order of four homologous regions in its mitogenome, while the majority other centipedes showed a D-E-B-C order (Figure 3). The F region was shorter and more conserved in all six homologous regions. However, there was an absence of the F region and a clearly shorter A region in the *Strigamia maritima* Leach, 1817 (KP173664.1) mitogenome. Alternatively, a large ratio of intergenic regions in the *S. maritima* mitogenome were identified, which was also previously reported (Chipman et al. 2014; Robertson et al. 2015). In the Lithobiomorpha order, the six homologous regions of *Bothropolys* sp. (AY691655.1) and *L. forficatus* (AF309492.1) were very similar for their length and the genomic location, while those in *C. longicornis* (NC_021403) were clearly different. Comparatively, in the Scolopendromorpha order, the lengths of these homologous regions across *S. mutilans*, *S. dehaani*, and *Scolopocryptops* sp. mitogenomes were conserved, though there was a clear rearrangement among them.

### Phylogenetic analysis

**Figure 4.**
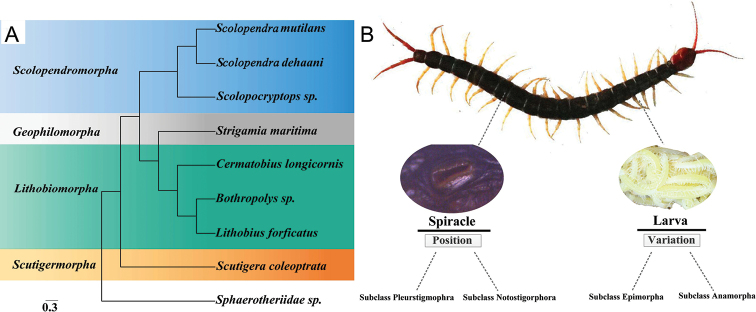
**A** Molecular phylogeny of eight centipede species based on Maximum Likelihood inference analysis of 13 protein-coding genes (PCGs) **B** Traditional morphological classification based on the position of spiracles and the variation of larvae.

The constructed ML tree is presented in Figure 4. As previously expected, *S. mutilans*, together with *S. dehaani* and *Scolopocryptops* sp., was placed in one group belonging to the Scolopendromorpha order. Moreover, our phylogenetic analysis suggested that the Scutigeromorpha order (*Scutigera coleoptrata*) was a sister group with the other three centipede orders, Scolopendromorpha, Geophilomorpha (*S. maritima*) and Lithobiomorpha (*C. longicornis*, *Bothropolys* sp., and *L. forficatus*). Our analysis further showed a close relationship between the orders Geophilomorpha and Lithobiomorpha, although the traditional morphological taxonomy suggested a potentially close relationship between the Geophilomorpha and Scolopendromorpha orders due to their shared trait of a stable segment number and lateral spiracles (Fernández et al. 2014).

The ω value can be used for revealing the constraints of natural selection (Tomoko 1995). Among our calculations, the ω value of 13 PCGs were all distributed around 0.004 (Suppl. material 1: Table S1), indicated a possibly purifying selection.

## Discussion

We sequenced and assembled the mitogenome of *S. mutilans*, a representative animal widely used in Chinese traditional medicine. The mitogenome is 15,011 bp in length, which is similar to the genome size of other known centipede mitogenomes, for example, 15,119 bp in *Scolopocryptops* sp. and 15,139 bp in *Bothropolys* sp. (Table 1). The variation of the Chilopoda mitogenome size was relatively conserved, which was consistent with that reported in Diplopoda, an animal class close to Chilopoda (Dong et al. 2016). The gene distribution was mainly presented on the plus strand of the *S. mutilans* mitogenome, and only four PCGs and two rRNA genes were located on the minus strand (Figure 1). This was consistent with other centipede species, like *S. maritima* and *S. dehaani* reported in previous studies (Robertson et al. 2015; Sun et al. 2018). Comparatively, the 13 PCGs in the *S. mutilans* mitogenome revealed a relatively low GC content, which was similar to that of *S. dehaani* (Figure 2).

Our study predicted nine mitogenomic SSRs, which can provide additional genetic marker information in molecular identification of centipede species (Table 3). Generally, the identification and genetic evaluation of centipede taxa depend on the variation presented in the cox1 gene region (Chen et al. 2013). However, when samples were investigated within species or between the closely related taxa, it is difficult to identify variation at individual or population level by only using the cox1 gene information (Kang et al. 2017). Comparatively, due to the relatively high mutation rate and the potentially neutrally evolutionary trajectory of SSR loci, they are widely used in animal genetic research under the species level, including assessing genetic diversity of wild populations, accelerating the progress of genetic selection, and molecular assistant breeding (Zhang et al. 2014). Our nine mitogenome SSRs were valued for future genetic research of samples from both *S. mutilans* and its closely related taxa.

We identified six homologous regions among the eight species’ mitogenomes, which revealed obviously genomic rearrangements, in particular between *S. mutilans* and some other centipedes (Figure 3). Genomic rearrangement is common and potentially randomly presented in animals’ mitogenomes (Chen et al. 2016). With the increase of mitochondrial genome data of animals, it is clear that rearrangements in mitogenomes are more a matter of sampling than a product of evolution (Boore 1999). For example, Negrisolo et al. (2003) found that it is less reliable to infer phylogenetic relationships based on gene order data in Arthropoda. Genomic rearrangements also occurred randomly among different orders in Hexapoda insects, which is not directly related to the evolution of groups (Cameron et al. 2006). Nevertheless, the observed mitogenomic rearrangements of Chilopoda taxa showed information about how genes move dynamically between different mitogenomes, which may be related to each individual gene evolutionary pattern.

Previous studies revealed alternative phylogenetic relationships of different centipedes by using different molecular datasets (Regier et al. 2008; Robertson et al. 2015; Fernández et al. 2016). With the obtained whole mitogenomic information of *S. mutilans* and the comparative analysis with other representative centipede taxa, our phylogenetic tree revealed a close relationship between *S. mutilans* and *S. dehaani*, which commonly belongs to the Scolopendromorpha order together with *Scolopocryptops* sp. (Figure 4). This was consistent with previous research (Lewis et al. 2005). However, at the order level, with increased two Scolopendromorpha samples, our analysis showed a closer relationship between Geophilomorpha and Lithobiomorpha, rather than between Geophilomorpha and Scolopendromorpha, which was slightly different to previous research (Robertson et al. 2015). Given the potentially dynamic evolutionary trajectory of different genes or between nuclear and mitochondrial genomes, this discordance may reflect the complex evolutionary history of these centipedes, including the possibility of a genetic admixture or adaptive radiations of these lineages in relation to morphological or functional specification in different geographical areas.

In conclusion, we successfully sequenced the complete mitochondrial genome of *S. mutilans* for the first time using next-generation sequencing, which will be valued for further studies in terms of the conservation, molecular identification, and evolutionary biology of diverse centipede species, improving the medicinal applications of *S. mutilans* and other closely related taxa.

## Supplementary materials

Supplementary material 1 Table S1 Explanation note The ω value of 13 PCGs. https://binary.pensoft.net/file/398381This dataset is made available under the Open Database License (http://opendatacommons.org/licenses/odbl/1.0/). The Open Database License (ODbL) is a license agreement intended to allow users to freely share, modify, and use this Dataset while maintaining this same freedom for others, provided that the original source and author(s) are credited.Chaoyi Hu, Shuaibin Wang, Bisheng Huang, Hegang Liu, Lei Xu, Zhigang Hu, Yifei Liu
